# Research on Image Mapping Spectrometer Based on Ultra-Thin Glass Layered Mapping

**DOI:** 10.3390/s24061763

**Published:** 2024-03-08

**Authors:** Pengwei Zhou, Yangfan Lv, Jiamin Zhou, Yuqi Zheng

**Affiliations:** College of Optical and Electronic Technology, China Jiliang University, Hangzhou 310018, China

**Keywords:** hyperspectral imaging, IMS, image mapper, ultra-thin glass, edge eating

## Abstract

The imaging quality of the Mapping Imaging Spectrometer (IMS) is crucial for spectral identification and detection performance. In IMS, the image mapper significantly influences the imaging quality. Traditional image mappers utilize a single-point diamond machining process. This process leads to inevitable edge eating phenomena that further results in noticeable deficiencies in imaging, impacting spectral detection performance. Therefore, we propose a manufacturing process for the image mapper based on ultra-thin layered glass. This process involves precision polishing of ultra-thin glass with two-dimensional angles, systematically assembling it into an image mapper. The surface roughness after coating is generally superior to 10 nm, with a maximum angle deviation of less than 3′. This results in high mapping quality. Subsequently, a principle verification experimental system was established to conduct imaging tests on real targets. The reconstructed spectrum demonstrates excellent alignment with the results obtained from the Computed Tomography Imaging Spectrometer (CTIS). We thereby validate that this approach effectively resolves the issues associated with edge eating (caused by traditional single-point diamond machining), and leads to improved imaging quality. Also when compared to other techniques (like two-photon polymerization (2PP)), this process demonstrates notable advantages such as simplicity, efficiency, low processing costs, high fault tolerance, and stability, showcasing its potential for practical applications.

## 1. Introduction

**Figure 1:**
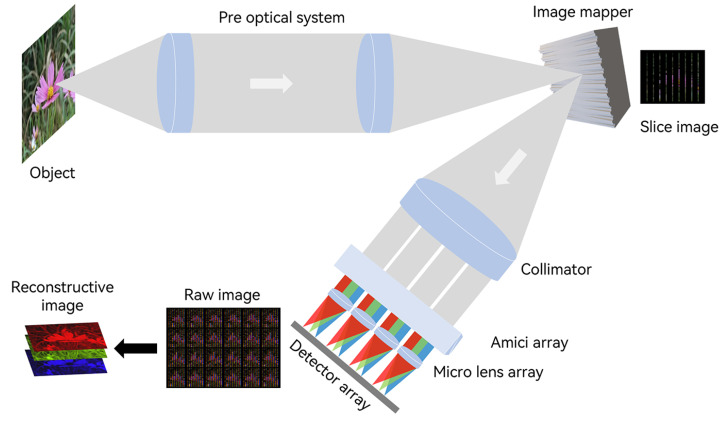
IMS system. Light from the object is collimated through a pre-optical system and imaged on an image mapper, then reflected in a sliced form into a collimating mirror. Subsequently, it is imaged on an image sensor through an Amici prism array and a micro-lens array. Finally, the three-dimensional spatial spectral data are reconstructed into a two-dimensional image.

Snapshot Imaging Spectrometers are widely used in various fields, such as remote sensing [1], biomedical imaging [2,3], and gas and environmental monitoring [4]. In snapshot imaging spectrometers, the Image Mapping Spectrometer (IMS) is characterized by its simple optical path, high spatial resolution, moderate spectral resolution, and high optical flux. It is considered as the optimal choice for field detection tasks, as recognized at the CTBTO Science and Technology 2015 conference [5]. Figure 1 shows that the IMS utilizes an image mapper to disperse the target in a sliced form on a two-dimensional plane. Spectral information is then filled in the blank spaces of these slices using a dispersive prism, allowing the acquisition of both spatial and spectral information. The image mapper is composed of multiple two-dimensional tilted mirror surfaces. It reflects the sliced images of the target into the sub-pupils of a micro-lens array, playing a vital role in determining the imaging quality of the IMS [6].

**Figure 2:**
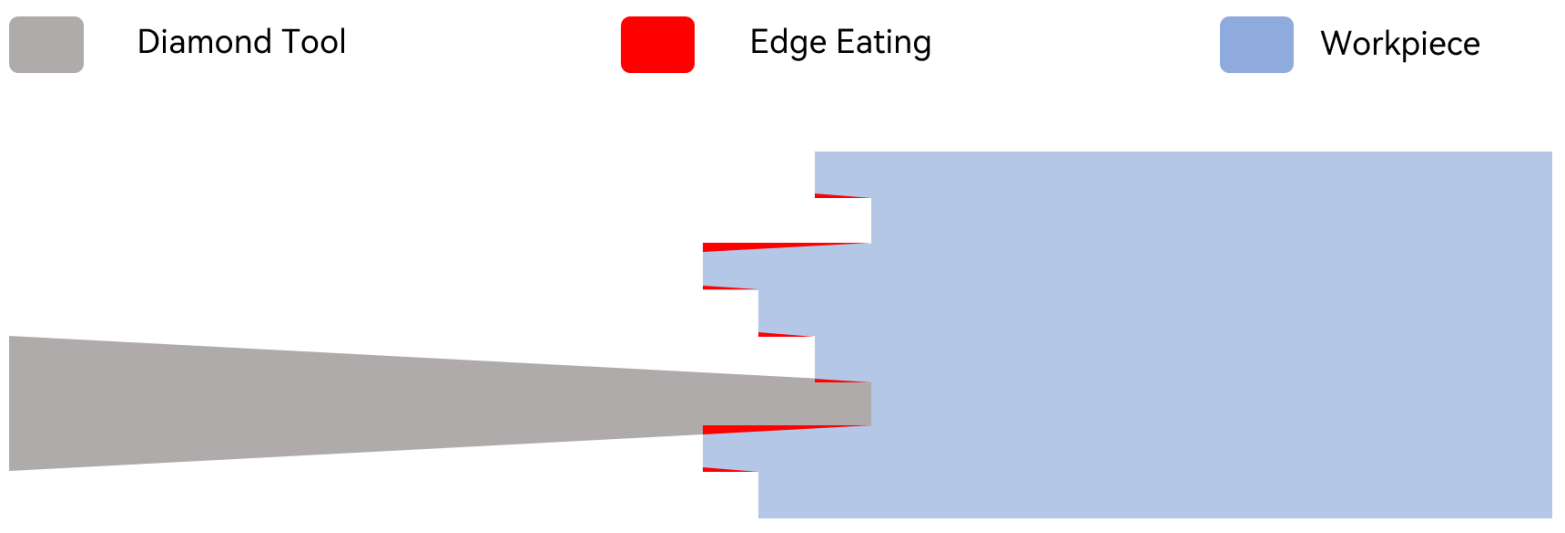
Single-point diamond machining technique. Since the angle of a diamond cutting tool cannot be reduced to 0°, it causes damage to adjacent mapping surfaces during processing.

In the early stages of application of IMS systems, image mappers primarily employed diamond machining [7]. Utilizing the raster fly-cutting technique based on high-precision single-point diamond machining, image mappers with 25 mapping angles, measuring 16 mm × 16 mm, were initially produced on high-purity aluminum. However, due to the inability to achieve a 0° cutting tool tip angle, the surface inevitably exhibits a certain degree of edge eating phenomenon [7], as depicted in Figure 2. Subsequently, the ruling method, also based on high-precision single-point diamond machining, was employed to create mappers with over 300 mapping surfaces. This approach aimed to enhance processing speed while minimizing edge eating dimensions by using diamond tools with smaller tip angles [8]. Nevertheless, the edge eating issue persisted, affecting the imaging quality. Additionally, specialized diamond tools and mappers still faced challenges, such as processing difficulties, high costs, and long processing cycles.

In recent years, researchers have explored various approaches to improve mapping quality and processing efficiency [9,10,11]. For instance, the use of a liquid crystal spatial light modulator (SLM) has been proposed as a replacement for traditional passive mappers, enhancing system control flexibility [12]. Some researchers have employed two-photon polymerization (2PP) to produce serrated image mappers [13], successfully addressing the edge eating problem. However, these methods still incur relatively high costs, with 2PP printing systems typically exceeding $500,000 [14]. Moreover, the required polymers often exhibit significant drawbacks, such as large thermal expansion coefficients (CTEs) and susceptibility to chemical degradation [15]. These issues result in deformation, delamination, and other problems in extreme environments, thereby compromising the operational performance of the spectrometer. Consequently, it is not suitable for tasks in extreme environments.

Astronomers have conducted research on one-dimensional image slicers to observe celestial bodies in the universe [16,17]. Glass processed through grinding and polishing techniques exhibits advantages, such as nanoscale surface roughness [18], interspaces between layered glass less than 5 μm [17], and low processing costs. In theory, it can be used to manufacture Image Slicer (IMS) devices that achieve two-dimensional mapping. This approach is promising for addressing edge eating issues in current single-point diamond machining [13]. However, there is currently no literature available that effectively demonstrates the production of a two-dimensional image slicer using this method due to significant differences in manufacturing processes between two-dimensional mappers and one-dimensional image slicers.

**Figure 3:**
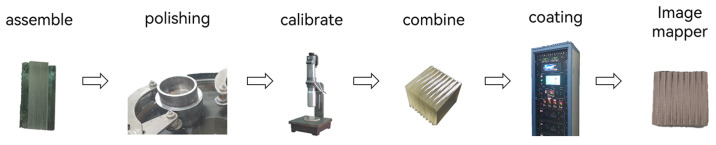
Ultra-thin glass image mapper processing workflow.

This paper attempts to use ultra-thin glass to manufacture an IMS system’s image mapper, as illustrated in Figure 3. The ultra-thin glass pieces are methodically assembled through the process of assembling multiple pieces of ultra-thin glass, performing precise polishing at specific angles, and repeatedly calibrating. Coating technology is employed to enhance its reflective properties, resulting in an image mapper based on ultra-thin glass.

## 2. A Manufacturing Process for the Image Mapper Based on Ultra-Thin Layered Glass

**Figure 4:**
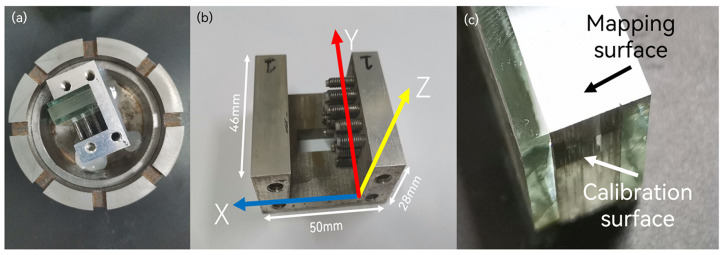
(**a**) Carrier plate and trim ring. The carrier plate is used for loading angle molds, and the trim ring is used to ensure stable grinding and polishing. (**b**) Ultra-thin glass angle mold. (**c**) Ultra-thin glass mapping surface and calibration surface.

To ensure optimal imaging quality, the mapping angles of the image mapper can be designed according to the size of a full-frame camera sensor, with a 2:3 ratio designed for 4 × 6, resulting in a total of 24 two-dimensional mapping angles. By utilizing the principle of diagonal mirroring, the 24 sets of angles to be processed are compressed into 12 sets, which improves the processing efficiency of the image mapper and reduces angle errors when processing multiple sets with the same angle, as shown in Figure 4a. Additionally, considering the size constraint of the carrier plate with an internal diameter of 80 mm, for precise grinding and polishing equipment, 12 molds for ultra-thin glass two-dimensional angles are designed with external dimensions of approximately 50 mm × 46 mm × 28 mm. It has an accommodation space of approximately 25 mm. This enables the simultaneous processing of nearly 100 pieces of ultra-thin glass while maintaining stability, thus improving the processing efficiency of the ultra-thin glass. As shown in Figure 4b, the z-x plane constrains the six long-side mapping angles (±0.015 rad, ±0.045 rad, ±0.075 rad) of the image mapper, while the z–y plane constrains the four short-side mapping angles(±0.015 rad, ±0.045 rad).

Soda-lime glass, known for its relatively low cost, makes a suitable material to validate the feasibility of processing ultra-thin glass. A size of approximately 0.1 mm × 25 mm × 20 mm for ultra-thin soda-lime glass can be selected for processing. Moreover, due to the inherent processing variations in actual ultra-thin glass, the final average thickness of the ultra-thin glass used is approximately 0.115 mm. However, this variation does not impact the validation of subsequent process feasibility. In the future, achieving a higher surface density can be explored by employing even thinner glass.

Due to the susceptibility of ultra-thin glass to breakage by uneven stress, it is essential to reinforce and protect its edges [19]. A buffering block, consisting of 3 mm thick soda-lime glass, is employed to disperse pressure and form a glass frame, preventing potential damage to the ultra-thin glass during the grinding process. Subsequently, measurements of the edge thickness at each vertex of the glass frame are conducted to ensure an overall thickness error of less than 20 μm. This ensures uniform stress distribution on the ultra-thin glass and minimizes gaps between individual pieces of ultra-thin glass. In addition to this, the ultra-thin glass is segmented into mapping and calibration surfaces, as shown in Figure 4c. While calibrating the mapping surface, adjustments are made to the calibration surface’s angles, aligning it perpendicular to the laminated plane. This facilitates the subsequent assembly of the ultra-thin glass layers.

**Figure 5:**
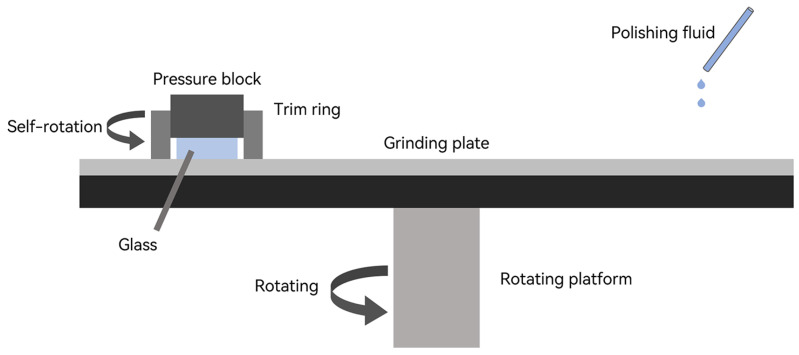
Ultra-thin glass polishing. Under the pressure of the pressing block and the wetting of the polishing fluid on the rotating platform, the ultra-thin glass realizes polishing.

The automatic grinding and polishing machine is an ideal piece of equipment for materials such as crystals, glass, and metals. It enables samples to undergo irregular motion on the grinding disc, ensuring uniform surface polishing without generating edge chamfer, as depicted in Figure 5. And compared with expensive 2PP printers [14] and the high-precision computer numerical control machine tools (CNC) required for diamond machining, the equipment cost of grinding and polishing is often relatively low. To enhance the efficiency and precision of the glass frame polishing process, a precision grinding and polishing machine (UNIPOL-1502) with three processing stations is used. During the polishing process, coarse polishing is initially performed using white corundum micro-powder (Dia = 5 μm), followed by fine polishing with ${\mathrm{CeO}}_{2}$ particles (Dia = 20 nm). The grinding angles of the glass frame are corrected through repeated measurements using an optical goniometer (Tianjin JJC7). The goniometer utilizes the principle of auto-collimation, combined with customized angle blocks, to achieve angle measurement. The processing of a single set of mapping angles takes approximately 8 h. And the entire procedure is iterated, including polishing the calibration surface of the glass frame, with a total processing time of around 40 h to obtain 12 sets of mapping angle glass frames.

**Figure 6:**
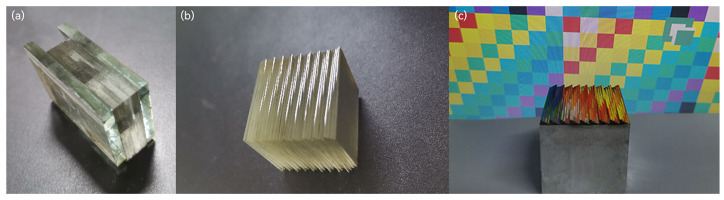
(**a**) Ultra-thin glass frame after polishing. (**b**) Bonded together. (**c**) Image mapper.

The processing techniques involving single-point diamond machining and 2PP printing often risk sample rejection due to errors in the machining of a specific reflective surface. In the proposed solution, a set of glass frames comprises nearly a hundred pieces of ultra-thin glass at the same angle, as shown in Figure 6a. The designed image mapper requires only 18 pieces for a single set of mapping angles. The surplus of ultra-thin glass in each set increases the fault tolerance during the layering process, allowing targeted replacement of error-prone pieces and effectively enhancing processing efficiency.

Subsequently, using the calibration surface as a reference, 24 mapping angles form one set of cycles. Nine cycles are assembled, as shown in Figure 6b, and vacuum deposition technology (RZD-500) is employed to coat the ultra-thin glass mapping surface with aluminum film, enhancing its reflection efficiency. This process results in a glass image mapper consisting of 216 pieces of ultra-thin glass, with approximate dimensions of 25.64 mm × 20 mm × 20 mm, and the average spacing between each glass piece is less than 5 μm, as depicted in Figure 6c.

The overall process takes approximately 50 h and supports simultaneous large-scale production across multiple devices and workstations. This represents a significant reduction in processing time compared to single-point diamond machining techniques, which may take weeks to produce a single mapper.

**Figure 7:**
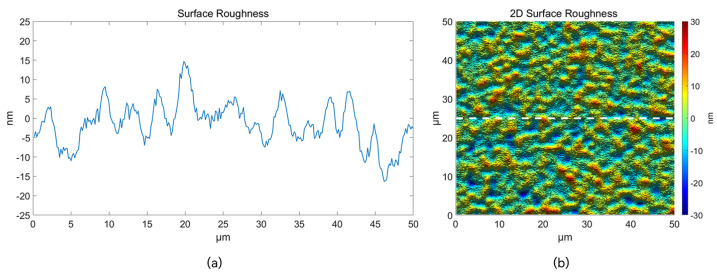
(**a**) One-dimensional horizontal roughness at dotted line position. (**b**) 2D Surface roughness. Where the white dotted line is the selected area in the two-dimensional plane.

To validate the coating quality on the mapper’s surface, the data of the mapping surface within a 50 μm × 50 μm field of view (0.015 rad, 0.015 rad) was measured using a white light interferometer (Talysurf CCI2000), as depicted in Figure 7. Due to the polishing process involving chemical etching and mechanical grinding, the two-dimensional surface exhibits relatively smooth undulations without distinct grooves. Its surface roughness measures at 5.65 nm, generally better than 10 nm, meeting the surface roughness requirements for the mapper [7]. The angles post-coating for the mapper are detailed in Table 1. By utilizing an aperture to constrain the field of view, the mapping angles for each mapping surface were calculated through pixel deviation. These values were then compared with the designed angles pixel positions to compute the error. The maximum angles error does not exceed $3^{\prime }$. Based on the above, Table 2 shows a brief comparison between different fabrication methods of the image mapper.

## 3. Primary Imaging Experiment

**Figure 8:**
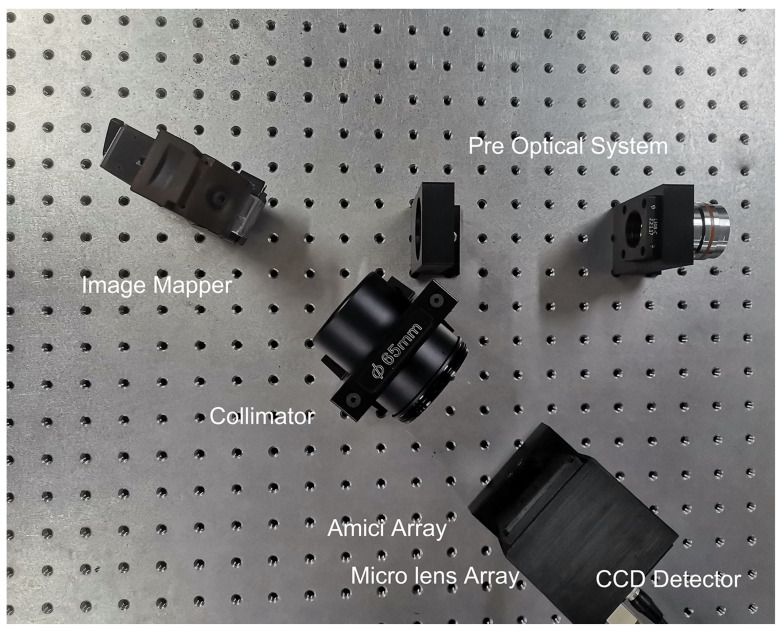
IMS experimental system includes Pre Optical System, Image Mapper, Collimator, Amici Array, Micro Lens Array and Image Detector.

To verify the imaging quality of the image mapper made of ultra-thin glass, an imaging experiment setup was constructed based on the IMS principle to test the imaging quality of the image mapper. The experimental setup mainly consists of a front lens group, image mapper, collimating lens, Amici prism array, micro-lens array, and image detector. The front lens group (2.5× optical relay), functioning as a 2.5× telecentric optical path, consists of a microscope objective (Zeiss Epiplan-Neofluar HD 2.5×/0.075 Objective) and a tube lens (165 mm Zeiss Tube lenses). After undergoing a single imaging reflection through the image mapper, the collimation is performed using an objective lens (Olympus MVPLAPO 1×), followed by dispersion using an Amici prism array, and then collimation using a micro-lens array (F.L. = 8 mm, Dia = 4.5 mm). Finally, imaging on a full-frame CCD (Vieworks VM-16M3, with a pixel count of 4872 × 3248, pixel size: 7.4 μm × 7.4 μm, photosensitive area size: 36 mm × 24 mm), is illustrated in Figure 8.

**Figure 9:**
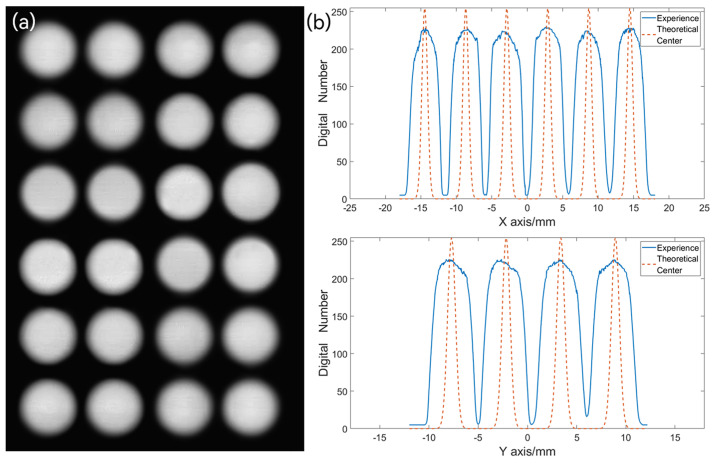
(**a**) Sub-pupils array. (**b**) Analysis of sub-pupils position and intensity information.

To assess the imaging quality of the image mapper, one can directly observe the sub-pupil images captured by the CCD camera. Using a white LED as a uniform illumination source, the light passes through a front collimating lens, images on the image mapper, reflects through multiple angles on the mapper, and is captured by the CCD camera sensor through a rear collimating optical path. The intensity differences among various sub-pupils can be directly observed, as depicted in Figure 9a. In this analysis, six sub-pupils at −0.045 rad and four sub-pupils at −0.075 rad are considered. Figure 9b displays the reflection intensity and coordinate positions of each sub-pupil. Evidently, the reflection intensity of each sub-pupil appears uniform, and there is good consistency between the actual and theoretical center positions of the sub-pupils. This observation further validates the accuracy of the mapper’s angle processing, confirming its precise angular alignment.

**Figure 10:**
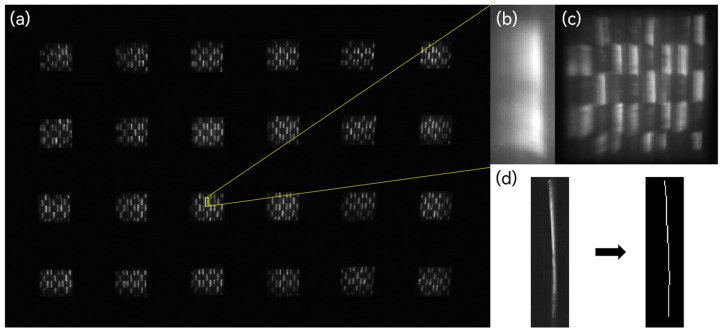
(**a**) IMS image. (**b**) Dispersion. (**c**) Single sub-pupil image. (**d**) Geometric transformation operations.

To evaluate the spectral image reconstruction performance of the IMS system, spectral image reconstruction tests were conducted using a white LED as the light source and a real image as the target, as shown in Figure 10. In the process of image reconstruction, the two-dimensional data (x, y) of the captured images need to be mapped to a three-dimensional spectral data cube (x_0_, y_0_, z_0_). The 24 mapping images were positioned according to the imaging sequence. However, due to image distortion, each positioned image may exhibit a certain degree of angular tilt. To address this issue, graphic transformation operations were employed, along with the identification of the mapping imaging boundary pixels using a slit, to reposition the mapping pixel locations.

Due to the designed spectral filling width of approximately 24 pixels, a grating monochromator (Zolix omni λ300) was employed to calibrate the dispersion spectrum. This calibration was essential for determining the mapping pixel positions corresponding to the dispersion spectrum and its associated spectral bands. The calibrated dispersion spectrum facilitates the subsequent reconstruction process by enabling the accurate mapping of information from each spectral band to the data cube for reconstruction.

## 4. Result Analysis

To validate the accuracy of spectral reconstruction, two reference points, A and B, were selected in real target images. The spectral reconstruction results from a Computer Tomography Imaging Spectrometer (CTIS) (HORIBA Verde) were used as a reference.

**Figure 11:**
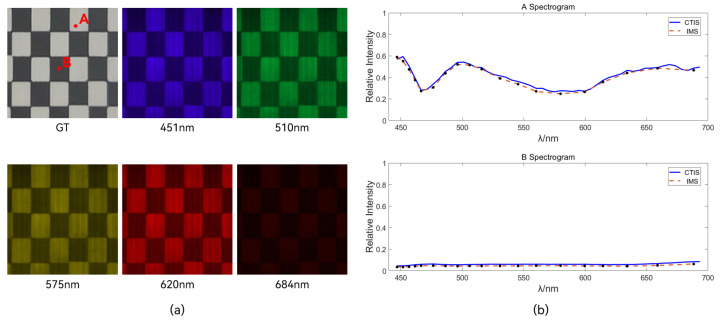
(**a**) Reconstructed image based on partial band pseudo-colored spectrum drawn by CIE1931, and two points A and B were marked on the real image (GT). (**b**) Comparison of spectral data of A and B points.

As shown in Figure 11b, it can be observed that the reconstructed spectral data from IMS exhibit high consistency with the spectral data reconstructed by CTIS within the visible light range. The spectral plot at reflection point A reveals the relative intensities of various spectral bands from the light source. In contrast, at reflection point B, the overall spectral intensity is lower due to the substantial absorption of energy. The reconstructed spectra effectively capture the trends in spectral changes, confirming the reliability of the IMS image reconstruction method and the imaging performance of the ultra-thin layered glass mapper. In Figure 11a, five characteristic spectral bands were selected from all reconstructed spectral images, and their pseudo-colored images were plotted according to CIE1931 standards [20]. However, due to differences in the non-ideal optical path and the quality of the reflective surface of the image mapper, there are subtle stripe shadows in the reconstructed images. The mentioned stripe artifacts can be effectively eliminated by optimizing the coating process and applying intensity compensation during the image reconstruction process [21]. We plan to address these issues and enhance the quality of image reconstruction in our future work.

It is noteworthy that traditional single-point diamond-machined mappers exhibit image loss due to the edge eating problem [10,22,23]. In contrast, the reconstructed images using an ultra-thin glass mapper do not display missing features, confirming the advantage of using ultra-thin glass to address the edge eating issue associated with traditional single-point diamond machining. This highlights the superiority of the ultra-thin glass mapper compared to traditional mapper fabrication approaches.

## 5. Conclusions

In summary, traditional IMS image mappers commonly employ a single-point diamond machining process, which is associated with issues, such as high processing costs, lengthy processing times, and low fault tolerance. Most crucially, it introduces edge eating problems in image mappers, resulting in a degradation of imaging quality. Therefore, we have developed an image mapper based on ultra-thin layered glass and have conducted imaging tests. The results indicate that this approach not only resolves the edge eating issues associated with traditional single-point diamond machining, but also improves imaging quality. It also offers advantages such as simplicity, efficiency, low processing costs, and high fault tolerance. In comparison to the emerging 2PP technology, the approach has advantages such as excellent material stability and lower processing costs, making it more suitable for tasks in extreme environments.

Future work will focus on optimizing the optical path, refining polishing and coating processes, and compensating for image reconstruction errors to further enhance the practical application value of IMS in remote sensing, detection and applications to other fields. 

## Figures and Tables

**Table 1:** Ultra-thin glass 2D angle.

Angle	Z-Y Plane			Z-X Plane		
Group	Design/rad	Measure/rad	Deviation (′)	Design/rad	Measure/rad	Deviation (′)
1	0.015	0.01484	0.550	−0.075	−0.07476	0.825
2	0.015	0.01513	0.447	−0.045	−0.04451	1.684
3	0.015	0.01542	1.444	−0.015	−0.01542	1.444
4	0.015	0.01484	0.550	0.015	0.01513	0.447
5	0.015	0.01484	0.550	0.045	0.04480	0.688
6	0.015	0.01454	1.581	0.075	0.07505	0.172
7	0.045	0.04422	2.681	−0.075	−0.07505	0.172
8	0.045	0.04451	1.684	−0.045	−0.04509	0.310
9	0.045	0.04422	2.681	−0.015	−0.01484	0.550
10	0.045	0.04509	0.310	0.015	0.01542	1.444
11	0.045	0.04451	1.684	0.045	0.04451	1.684
12	0.045	0.04480	0.688	0.075	0.07534	1.169

**Table 2:** Comparison of 2PP, ultra-thin glass layered and diamond machining.

	Diamond Machining	Ultra-Thin Glass Layered	Two-Photon Polymerization
Fabrication time	2–3 weeks	≈50 h	≈78 h
Spatial density	70–80 μm	100 μm	2–3 μm
Precision of angles	<$7^{\prime }$	<$3^{\prime }$	<$4^{\prime }$
Edge eating	Yes	No	No
Processing cost	High	Low	High
Fault tolerance	Low	High	Low
Material stability	stable	stable	Large thermal expansion coefficients

## Data Availability

Data underlying the results presented in this paper are not publicly available at this time but may be obtained from the authors upon reasonable request.
